# Genomic Regions Associated With Seed Meal Quality Traits in *Brassica napus* Germplasm

**DOI:** 10.3389/fpls.2022.882766

**Published:** 2022-07-14

**Authors:** Gurleen Bhinder, Sanjula Sharma, Harjeevan Kaur, Javed Akhatar, Meenakshi Mittal, Surinder Sandhu

**Affiliations:** Oilseeds Section, Department of Plant Breeding and Genetics, Punjab Agricultural University, Ludhiana, India

**Keywords:** antioxidants, antinutritional traits, *Brassica napus*, cluster analysis, genome-wide association study, seed meal quality

## Abstract

The defatted *Brassica napus* (rapeseed) meal can be high-protein feed for livestock as the protein value of rapeseed meal is higher than that of the majority of other vegetable proteins. Extensive work has already been carried out on developing canola rapeseed where the focus was on reducing erucic acid and glucosinolate content, with less consideration to other antinutritional factors such as tannin, phytate, sinapine, crude fiber, etc. The presence of these antinutrients limits the use and marketing of rapeseed meals and a significant amount of it goes unused and ends up as waste. We investigated the genetic architecture of crude protein, methionine, tryptophan, total phenols, β-carotene, glucosinolates (GLSs), phytate, tannins, sinapine, and crude fiber content of defatted seed meal samples by conducting a genome-wide association study (GWAS), using a diversity panel comprising 96 *B. napus* genotypes. Genotyping by sequencing was used to identify 77,889 SNPs, spread over 19 chromosomes. Genetic diversity and phenotypic variations were generally high for the studied traits. A total of eleven genotypes were identified which showed high-quality protein, high antioxidants, and lower amount of antinutrients. A significant negative correlation between protein and limiting amino acids and a significant positive correlation between GLS and phytic acid were observed. General and mixed linear models were used to estimate the association between the SNP markers and the seed quality traits and quantile-quantile (QQ) plots were generated to allow the best-fit algorithm. Annotation of genomic regions around associated SNPs helped to predict various trait-related candidates such as *ASP2* and *EMB1027* (amino acid biosynthesis); *HEMA2, GLU1*, and *PGM* (tryptophan biosynthesis); *MS3, CYSD1*, and *MTO1* (methionine biosynthesis); *LYC* (β-carotene biosynthesis); *HDR* and *ISPF* (MEP pathway); *COS1* (riboflavin synthesis); *UGT* (phenolics biosynthesis); *NAC073* (cellulose and hemicellulose biosynthesis); *CYT1* (cellulose biosynthesis); *BGLU45* and *BGLU46* (lignin biosynthesis); *SOT12* and *UGT88A1* (flavonoid pathway); and *CYP79A2, DIN2*, and *GSTT2* (GLS metabolism), etc. The functional validation of these candidate genes could confirm key seed meal quality genes for germplasm enhancement programs directed at improving protein quality and reducing the antinutritional components in *B. napus*.

## Introduction

Rapeseed (*B. napus* L.) is an economically important group belonging to the family *Brassicaceae* that possesses high acreage of 36.24 million hectares and production of 73.16 million metric tons (MMT) during 2020–2021 and ranks the third largest sources of vegetable oil all over the world (Yang et al., 2019; USDA, 2021). The biggest rapeseed producing countries in 2019/2020 were Canada (19 MMT), China (13.1 MMT), and India (7.7 MMT); however, the European Union produced 16.83 MMT of rapeseed (Statista, 2021). Rapeseed is widely cultivated throughout the world as a source of oil and protein for food and feed purposes. The portion left after oil extraction from seeds of rapeseed is called as meal or cake as per the residual oil content in it. Both meal and cake are rich in protein (32–48%) (Sadeghi and Bhagya, 2009) but differ in their oil content which is 1–4% in the meal (Klein-Hessling, 2007) and 10–14% in cakes (Mahoonak and Swamylingappa, 2007). World production of rapeseed meals in 2020/2021 was 41.20 MMT, which was higher than in 2019/2020 (39.45 MMT) (USDA-FAS, 2021). Interestingly, improved oilseed production would also help in raising meal production in the forecast year by 5.2% to a total of 17 MMT (USDA, 2021).

Due to its low-volume, high-cost, high-quality protein, residual oil, and energy values, seed meal of *Brassica* is mainly streamlined into formulation of feed for dairy cattle, swine, poultry, and farmed fish (Wanasundara, 2011). The seeds of *B. napus* contain around 25–30% seed storage proteins, which are predominantly composed of cruciferin (60%), napin (20%), and other minor proteins such as oleosins and lipid transfer proteins (Gehrig et al., 1996). The richness in minerals, vitamins, well-balanced amino acid composition, high levels of sulfur containing amino acids (cysteine, methionine in napin), and efficient protein utilization in humans showed that it can be rated as a high-quality protein, compared to egg and milk proteins (Fleddermann et al., 2013). Seeds of *B. napus* also reserve carotenoids (5.34 μg/g), which possess antioxidant properties that scavenge oxygen radicals and have been accounted to decline the incidence of cardiovascular disease and cancers (Yu et al., 2008). The results of few studies have shown that quantitative trait loci (QTL) for seed oil and protein content are closely linked, and there is a negative correlation between protein and oil content (Grami et al., 1977; Gül et al., 2003). These findings are not surprising as both protein and oil compete for the same basic substrates in the biochemical pathway and therefore must be partly controlled by the same genes. Also, the presence of carotenoids protects seeds against deterioration and aging and promotes seed germination (Howitt and Pogson, 2006). Despite its high nutritional importance, rapeseed meal usage as an ingredient of food and feed is narrow due to the presence of high amount of some antinutritional compounds *viz.*, crude fiber (4.56% in yellow seeded meal and 8.86% in black seeded meal) (Jiang et al., 2015), phenolics (38.50–63.95 mg/g) (Yang et al., 2015), sinapine (7–13 mg/g) (Matthäus and Zubr, 2000), glucosinolates (GLSs) (15.49–139.09 μmol/g) (Sen et al., 2018), phytic acid (2–4% in the defatted meal, and 5–7% in the protein concentrates) (Sashidhar et al., 2020), tannins (2.71–3.91%) (Fenwick et al., 1984), etc., which cannot be quickly metabolized and have negative effect on animal health. As a consequence, a large amount of rapeseed meal remains unused and thus becomes a waste product (Aukema and Campbell, 2011).

Glucosinolates present in the Brassica seeds are hydrolyzed to pungent and biologically active isothiocyanates that have negative thyrogenic effects on animals (Walker and Booth, 2001). A modified improved quality of rapeseed developed in Canada has been named “canola” or “double low” variety, for its low content of erucic acid (<2%) in oil and glucosinolates (<30 μmol/g) in seed meal residue which have been fairly successful and considered excellent for food and feed purpose. The meal can thus be used as a protein supplement (Tripathi and Mishra, 2007). The pathways of amino acid and glucosinolate biosynthesis share common enzymes; therefore, perturbation of glucosinolate in double low varieties could affect the level of napin (Field et al., 2004; Nesi et al., 2008). The level of napin and cruciferin in seeds also affects the functionality of canola protein products, including solubility, emulsifying ability, and heat-induced gel formation. Moreover, the phenolic compounds are the major contributors to the dark color and astringent, bitter taste of the meal which is found in the seed coat and cotyledons (Hannoufa et al., 2014). The rich phenolic acid in rapeseed is sinapic acid (3, 5-dimethoxy-4-hydroxycinnamic acid). Sinapine, its choline ester, accounted for ~80% of all phenolics, in *B. napus* seed (Wanasundara, 2011). Sinapine can complex with meal proteins and reduce their bioavailability and digestibility (Nesi et al., 2008). Hydrolysable and condensed tannins in rapeseed meal are the secondary compounds with antinutritional properties (Chung et al., 1998). *In vivo* experiments showed that hydrolysable tannins degrade into smaller compounds and injure both liver and kidney in ruminants, rodents, and poultry (Tosi et al., 2013; Bilić-Šobot et al., 2016). Phytates (myo-inositol hexaphosphoric acid) is the principal storage form of phosphorus which accumulates as insoluble crystals known as globoids in protein storage vacuoles of *Brassicaceae* seeds (Madsen and Brinch-Pedersen, 2020). Due to its ability to bind important dietary minerals (Ca, Zn, and Fe) as well as proteins and starch, it is considered as an antinutrient. Fiber fraction is linked with low digestibility and bioavailability of meal protein in feeds of animals. Total meal digestibility indicates a negative correlation with the hull and the lignin content of seed (Wanasundara, 2011). Approximately, one-third of *B. napus* meal is represented by dietary fiber with a significant amount of fiber being in the form of indigestible lignin.

Emphasis on reducing the amount of these antinutritional compounds in rapeseed meal must be given for addressing food security issue. Understanding the genetics of these traits is important for developing high-quality rapeseed meal for food and feed purpose. Genome-wide association study (GWAS) has become an effective and powerful tool for the dissection of loci associated with complex traits in the crop genomes especially for polyploids such as *B. napus*. GWAS determines the historical recombination between the trait of interest and single-nucleotide polymorphism (SNP) markers. If recombination between two loci is less frequent than expected for the unlinked regions, then the loci are known to be in linkage disequilibrium (LD). Many studies document identification of molecular markers associated with various seed quality traits such as protein (Akhatar et al., 2020), glucosinolates (Qu et al., 2015; Tan et al., 2022), oil (Xiao et al., 2019), fatty acids (Gacek et al., 2017; Tang et al., 2019; Yao et al., 2020), and acid detergent lignin (Wang and Qin, 2017) in Brassica using GWAS approach. However, scarce information is available with Brassica breeders regarding molecular markers associated with other important seed meal quality traits mentioned above which led to the lack of understanding of the genetic systems underlying the biosynthesis of these key nutrients and antinutrients.

Keeping this in viewpoint, efforts have been made to screen the sequenced diversity set of 96 *B. napus* accessions for seed meal quality traits and to identify the genomic regions associated with these traits using GWAS.

## Materials and Methods

### Seed Material, Defatting, and Phenotyping

*B. napus* fixed diversity set, comprising of 96 accessions, constituted the experimental materials for the present investigations. Each genotype was sown in paired rows of 2 m row length at a row-to-row and plant-to-plant spacing of 45 and 10 cm, respectively, in alpha lattice design with two replications at PAU, Ludhiana. The germplasm set was evaluated for seed meal quality traits during 2019–2020. The dry mature ground seeds of each sample were defatted using conventional Soxhlet extraction apparatus. A quantity of the dried sample (4 g) was put into the thimble, and the materials were continuously extracted for 6–7 h using petroleum ether (60–80°C) as a solvent. Further, the thimble was removed and the defatted seed meal was allowed to dry in an oven at 60°C. The drying process was repeated until a constant weight was obtained. Defatted meal was then stored in the zip lock packets at 4°C till further biochemical analysis. The concentration of ten seed quality traits *viz.*, crude protein, methionine, tryptophan, total phenols, β-carotene, glucosinolates, phytate, tannin, sinapine, and crude fiber content in seed meal were estimated using the standard procedures mentioned below.

#### Crude Protein Content

Crude protein was estimated using MicroKjeldahl nitrogen method (McKenzie and Wallace, 1954). Sample (0.2 g) was digested with conc. sulfuric acid (10 ml) and 2 g of catalyst mixture (CuSO_4_.5H_2_O and K_2_SO_4_ in the ratio of 1: 10) to convert organic nitrogen to ammonium sulfate in solution followed by decomposition of ammonium sulfate with sodium hydroxide. The released ammonia was distilled into 2% boric acid. The nitrogen from ammonia was deduced from titration of the trapped ammonia with 0.1NHCl using dye solution (0.3 g bromocresol, 0.2 g methyl red in 400 ml of 90% ethanol). The percent nitrogen obtained was multiplied by the general factor 6.25 to give the percent crude protein.

#### Methionine Content

Methionine content was estimated by using method of Horn et al. (1946). About 1 g of dried seed meal sample was weighed and transferred into a 50-ml conical flask containing 2.5N HCl and autoclaved for 1 h at 121°C and 15 psi. A pinch of activated charcoal was added to the hydrolysate and heated to boil to decolorize the extract and was then filtered when hot. The volume of the filtrate was made to 25 ml with double distilled water. This filtrate (10 ml) was then transferred to a flask containing 3 ml of 5N and 0.1 ml of 10% sodium nitroprusside. After 10 min, 2 ml of 3% glycine solution was added and again after another 10 min, 4 ml of o-phosphoric acid was added and shaken vigorously. The intensity of red color was read at 520 nm. Methionine content was calculated by extrapolation on the standard graph prepared using 0.2 mg to 3 mg of methionine. Methionine content was expressed as g/100 g protein.

#### Tryptophan Content

Tryptophan content was determined according to Spies and Chambers (1949) method. To powdered seed meal sample (50 mg), 30 mg of p-dimethylaminobenzaldehyde (PDAB) and 10 ml of 19N H_2_SO_4_ were added in a flask which was then kept at room temperature in dark place for 12 h. Aliquot is centrifuged at 5,000 rpm for 30 min, and to it, 0.1 ml of 0.045% NaNO_2_ solution was added. After an incubation of 15 min, the color developed was read at 454 nm on spectrophotometer and calculated the content of tryptophan using standard curve for tryptophan (40–200 μg). Tryptophan content was expressed as g/100 g protein.

#### β-carotene

Beta-carotene content was assessed according to the approved methods of AOAC (1980). About 1 g of fine defatted sample was taken in glass vials, and 10 ml of water saturated butanol (WSB) (8 butanol: 2 water v/v) was added. The vials were closed tightly and mixed vigorously for 1 min and kept overnight (18–20 h) at room temperature under dark for complete extraction of β-carotene. Next day, the vials were slightly shaken again, and the extract was filtered through Whatman No. 1 filter paper. The optical density of the filtrate was measured at 440 nm on spectrophotometer. Pure WSB was used as blank. The β-carotene content was calculated from calibration curve from known amount of β-carotene and expressed as μg/g. Standard solution of β-carotene was prepared in WSB at the concentration of 5 μg/ml.

#### Total Phenol Content

Total phenol content was determined by following the method of Swain and Hillis (1959). Homogenization of 0.25 g defatted dried sample was done in 10 ml of 80% methanol and refluxed on water bath for 2 h at temperature 70–75°C. The methanolic extract was pooled after refluxing, and volume was made to 10 ml by washing with 80% methanol. The methanolic extract (0.5 ml, 50 g/ml) was mixed with Folin–Ciocalteu reagent (0.5 ml) and shaken thoroughly. After 5 min, saturated solution of Na_2_CO_3_ (1 ml) was added. After an hour of incubation at room temperature, the absorbance of blue color was read in a spectrophotometer at 760 nm against the blank. The blank was prepared from water and reagent only. The concentration of the total phenols (mg/g GAE) was calculated from the standard curve prepared using gallic acid (10–100 μg) (Thomas Baker Chemicals Private Limited, India).

#### Sinapine Content

Sinapine content was determined using method by Kolodziejczyk et al. (1999). Defatted meal (1.5 g) was extracted three times with 35 ml of methanol by refluxing for 30 min. Methanol extracts were combined and evaporated transferred to a 100-ml volumetric flask, and the final volume was adjusted to 100 ml with methanol. For measurement, 100 μl of solution was diluted to 10 ml with methanol and the absorbance was recorded at 330 nm. Sinapine content in the meal was calculated from the formula:

% Sinapine = (2.184 × Absorbance ×10)/ Sample Wt. [g].

#### Phytic Acid

Phytic acid content in seed meal was determined by following the method of Haug and Lantzsch (1983). Seed meal (0.2g) sample was homogenized in 25 ml of 0.2N HCl and was shaken continuously for 3 h on a mechanical shaker at room temperature. The extract was filtered through Whatman paper. An aliquot of 0.5 ml of this extract was pipetted into a test tube, and total volume was made to 1.4 ml with distilled water. Further, 1 ml of ferric ammonium sulfate or ammonium ferric (III) sulfate (FAS) solution (0.2 g of FAS was dissolved in 100 ml of 2N HCl and volume made up to 1,000 ml with distilled water) was added and the contents were stirred and heated for 30 min in boiling water bath. About 1 ml of aliquot was transferred to another test tube to which 1.5 ml of 2, 2'-bipyridine solution (1 g of 2, 2'-bipyridine in 1 ml of thioglycolic acid dissolved in distilled water and volume made to 100 ml) was added. The tubes were shaken well and the color intensity was read at 519 nm against distilled water as a blank in spectrophotometer. The concentration of the phytic acid (mg/100 g) was calculated from the standard curve prepared using sodium phytate (40–200 μg).

#### Crude Fiber

Crude fiber content was estimated following the AOAC (1990) protocol. Moisture and fat-free meal sample (1 g) was boiled in 100 ml of 1.25% H_2_SO_4_ solution for 1 h under reflux. The boiled sample was washed in several portions of hot water using 2-fold cloth till it becomes acid-free. The sample was transferred to the same beaker and boiled again in 100 ml of 1.25% NaOH for another 1 h under the same condition. To make alkali-free, the residue was washed in several portions of hot water and was allowed to drain dry before being transferred quantitatively to a weighed crucible (w_o_) where it is dried to constant weight (w_1_) at 100°C. Constant weighed crucible (w_1_) was then ignited in muffle furnace where it was burnt, only ash was left of it. The weight of the fiber was determined by difference and calculated as


$$\begin{array}{l} Crude\;fiber\;\left(\%\right)\;=\;\frac{\left(w_{1}\;-\;w_{o}\right)\;-\;\left(w_{2}\;-\;w_{o}\right)}{\text{Weight of sample}}\;\times \;100 \end{array}$$

#### Tannin Content

Tannin content estimation was conducted following Price et al. (1978) protocol. Defatted sample (0.2 g) was homogenized using 2 ml of reagent A (2.8% conc. HCl in methanol). Contents were vortexed for 20 min at room temperature and then centrifuged at 10,000 rpm for 10 min. The pellet was discarded and the 0.5 ml of supernatant was mixed with 2.5 ml of reagent C [1% vanillin in reagent B (22.2% of conc. HCl in methanol)] and incubated at 30°C for 20 min. Absorbance of the mixture was read at 500 nm on the spectrophotometer. Standard curve was prepared using catechin as standard in the range of 10–100 μg.

#### Glucosinolates

The determination of seed meal glucosinolates was performed as described by Kumar et al. (2004). About 500 mg seed was warmed at 100°C for 1 h in the oven to deactivate myrosinase. Seeds were then grounded and defatted three times using 30 ml petroleum ether each time. The residue was then dried in an oven at 100°C for 10 min. About 200 mg of fat-free sample was taken, and 0.3 ml of 60% warm methanol was added to it to deactivate the myrosinase enzyme. Tubes were then heated in water bath at 80°C for 10 min. Make sure that ethanol evaporates completely. About 4 ml of distilled water was added to it and tubes were heated further at 80°C for 15 min. The extract was centrifuged in a tube, and then, 40 μl of the supernatant was taken in other test tubes in duplicates. After addition of 4 ml of 0.2 mM Na_2_PdCl_4_ reagent, it was kept at room temperature for 1 h, and optical density reading (μmoles/g defatted meal) was recorded at 405 nm on the spectrophotometer. Sinigrin (16–83.0 μg) was used as standard.

### Statistical Analysis

Analysis of variance (ANOVA), coefficient of variance (CV) and best linear and unbiased predictors (BLUPs) were computed using SAS 9.3 (SAS Institute Inc.) and R software version 4.1.2 (https://www.r-project.org/).

### Diversity Analysis

D^2^ analysis is an important multivariate distance matric method to evaluate the genetic diversity and selection of parental material for the breeding programs based on the traits measured. It is based on measuring the distance between a point and a distribution. Mahalanobis D^2^ analysis was conducted using WINDOSTAT 8.0 cluster analysis tool using the Tocher's method (Rao, 1952).

### Genome-Wide Association Study

Genome-wide association study was conducted to find associations between SNPs and seed meal quality traits across the 96 diversity fixed *B. napus* accessions. Genotype by sequencing-based genome assembly, SNP data, and population structure of 96 diversity fixed set of *B. napus* was available from Pal et al. (2021) which was used to identify SNPs significantly associated with seed meal quality traits. The BLUP's value for the target parameters from each accession and SNP markers (77,889) were analyzed using software Genome Association and Prediction Integrated Tool (GAPIT) version 3 (Lipka et al., 2012; Wang and Zhang, 2021). Manhattan plots were plotted using R package “CMPlot.” We compared three different algorithm models for their capacity to fit the data: general linear model (GLM), mixed linear model (MLM), and fixed and random model circulating probability unification (farm CPU). The choice of the best model was based on quantile-quantile plot (Q-Q plot), by plotting their quantiles against each other. The association between SNPs and traits was assessed based on the –log_10_(*p*) value of each SNP, and the expected *p*-values were used for the selection. SNPs with an arbitrary value of –log_10_(*p*) ≥ 3 were considered as significant, and allelic effect estimates were calculated for them.

#### Gene Prediction

The region of 50-kb up/downstream of the associated SNP/SNPs was scanned for identifying candidate genes using *B. napus* reference genome. The predicted gene and its orthologous sequence were then annotated by Basic Local Alignment Search Tool (BLAST) analysis against *Arabidopsis thaliana* database using Blast2GO Pro tool (Conesa and Götz, 2008). Functions of the possible candidate genes were validated from NCBI (https://www.ncbi.nlm.nih.gov/) to determine their relevance for biochemical traits in question.

#### Gene Pathway

GeneMANIA is a user-friendly prediction web server (https://genemania.org/) which analyze the input gene list and priorities them for their function and fit them into possible networks based on their probable interactions or co-expressions, etc. (Warde-Farley et al., 2010). It also extends the query gene list with genes that may be functionally similar or associated in the same network based on the available genomic and proteomic data. We used this tool to find the associations within significant SNPs predicted for the seed meal quality traits.

## Results

### Phenotypic Variation in Seed Quality Traits

**Figure 1:**
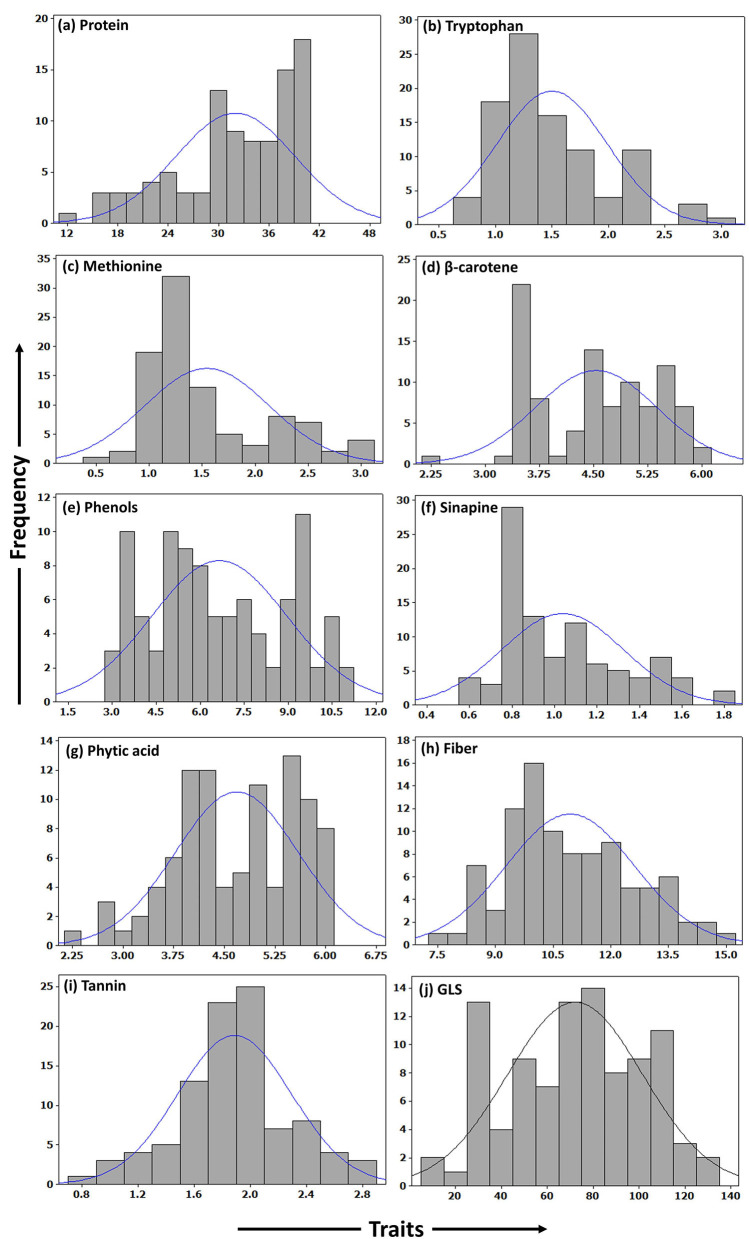
Frequency histograms showing distribution of quality traits in seed meal of B. *napus* accessions. **(a)** Protein. **(b)** Tryptophan. **(c)** Methionine. **(d)** β-carotene. **(e)** Phenols. **(f)** Sinapine. **(g)** Phytic acid. **(h)** Fiber. **(i)** Tannin. **(j)** GLS.

A total of ten seed meal quality traits *viz.*, crude protein, limiting amino acids (methionine and tryptophan), β-carotene, phenols, sinapine, phytic acid, crude fiber, tannins, and glucosinolate content were measured for three replications in *B. napus* diversity panel comprising 96 accessions. As displayed in Table 1, the results indicated that there were abundant phenotypic variations in 96 *B. napus* diversity panel, and all the seed meal quality traits followed a normal distribution (Figure 1), which benefited the dissection of the genetic architecture of the seed. Frequency distribution graph depicted that majority of genotypes had crude protein, methionine, tryptophan, β-carotene, phenols, sinapine, phytic acid, crude fiber, tannin content, and glucosinolates in the range of 38–40%, 1.0–1.5 g/100 g protein, 1.0–1.5 g/100 g protein, 3.3–3.5 μg/g, 9.3–9.7 mg/g GAE, 0.8–0.9%, 5.3–5.7%, 9.5–10.5%, 1.9–2.1%, and 75–85 μmoles/g defatted seed meal, respectively (Figure 1). The glucosinolate content, which varied from 12.27 to 128.31 μmoles/g defatted seed meal with an average of 72.18 μmoles/g defatted seed meal, had the maximum coefficient of variation of 40.79%, whereas crude fiber, which varied from 7.70 to 14.90% with an average of 10.94%, had the lowest coefficient of variation of 15.22%. Out of 96 *B. napus* accessions, eleven genotypes, namely, PN-45-1, FAN-628, VCN-9, OCN-106, CHARLTON, EC-609305, OCN-69, PN-47-1, BCN-16, ZY-008, and PN-87-3 showing high-quality protein, richness in the antioxidants along with less amount of antinutritional compounds were selected among all graded genotypes. Crude protein content showed a strong negative correlation with limiting amino acids *viz.*, methionine (*p* = −0.734) and tryptophan content (*p* = −0.739); however, the two limiting amino acids depicted a positive correlation (*p* = 0.613) between them. Apart from this, the glucosinolate content was positively associated (*p* = 0.290) with phytic acid (Table 2). Overall, the *B. napus* seed meal quality traits exhibited significant genetic variations, and it was suitable for association analysis.

**Table 1:** Phenotypic variation for ten seed meal quality traits in B. napus diversity panel.

**Trait**	**Min**.	**Max**.	**Mean ±SD**	**CV**
Protein (%)	11.67	39.38	32.00 ± 7.12	22.26
Methionine (g/100 g protein)	0.53	3.00	1.54 ±0.59	38.19
Tryptophan (g/100 g protein)	0.73	3.01	1.50 ±0.49	32.54
β-carotene (μg/g)	2.35	5.99	4.53 ±0.84	18.48
Phenols (mg/g GAE)	3.01	11.17	6.65 ±2.31	34.69
Sinapine (%)	0.56	1.81	1.04 ±0.29	27.55
Phytic acid (%)	2.35	6.00	4.68 ±0.91	19.42
Crude Fiber (%)	7.70	14.90	10.94 ±1.67	15.22
Tannin (%)	0.77	2.72	1.89 ±0.41	21.51
Glucosinolates (μmoles/g)	12.27	128.31	72.18 ±29.44	40.79

*PTN, protein; AOX, antioxidants; ANU, antinutrients; Min., minimum; Max., maximum; SD, standard deviation; CV, coefficient of Variation which was estimated as the ratio of the standard deviation to the mean of all accessions*.

**Table 2:** Pearson correlation coefficients for seed meal quality traits.

	**Phenols**	**β-carotene**	**Crude protein**	**Methionine**	**Tryptophan**	**Phytic acid**	**Fiber**	**Tannin**	**GLS**	**Sinapine**
Phenols	1.000	0.160	−0.165	0.039	0.038	0.117	−0.057	−0.043	0.062	−0.010
β-carotene		1.000	0.004	−0.043	−0.024	−0.074	0.053	−0.031	−0.030	−0.067
Crude protein			1.000	−0.734***	−0.739***	−0.138	0.026	0.146	−0.061	−0.030
Methionine				1.000	0.613***	0.135	−0.030	−0.120	0.168	−0.004
Tryptophan					1.000	0.135	0.055	−0.023	0.039	0.061
Phytic acid						1.000	−0.196	0.090	0.290**	−0.059
Fiber							1.000	0.101	0.004	−0.066
Tannin								1.000	0.130	0.070
GLS									1.000	0.028
Sinapine										1.000

Trait pairs affecting quality traits in B. napus diversity panel. *** and ** Significance levels at p value 0.001 >0.337 and 0.01 >0.267 respectively.

### Diversity Analysis

Based on D^2^ analysis, all the 96 accessions were grouped into ten diverse clusters. Clusters 2 and 4 had majority of genotypes (17). From a total number of 10 traits measured, maximum contribution of 84.7% was observed for the glucosinolates followed by the contribution of 13.3% by crude protein content. The inter-cluster distance ranged from 183.0 (clusters 6 and 8) to 8,521.1 (clusters 4 and 9). The intra-cluster distances varied from 48.4 (cluster 8) to 208.6 (cluster 10). The cluster diagram based on eucledian^2^ distance is shown in Supplementary Figure S1.

### Marker Trait Associations Identifies Potential Candidate Genes Regulating Quality Traits in *B. napus* Seed Meal

**Figure 2:**
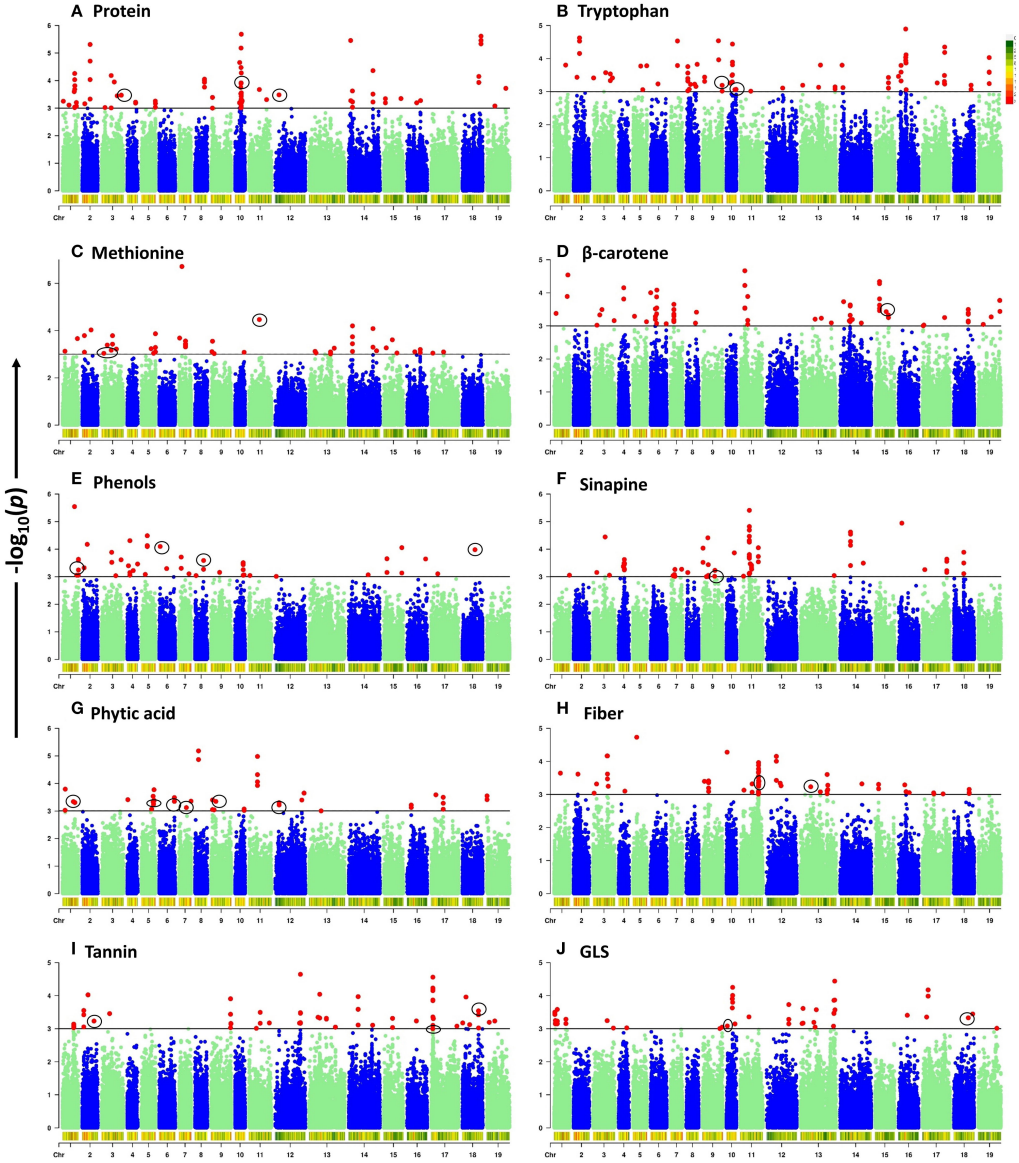
Manhattan plots of genome-wide association analysis for ten seed meal quality traits. -log_10_(*p*) values are plotted against position on each chromosome. Black lines at -log_10_(*p*) = 3 indicate the genome-wide significance threshold. SNPs with -log_10_(*p*) ≥ 3 were considered as significant. Chromosomes 1–10 represent A genome mentioned as A01–A10 and chromosomes 11–19 represent C genome mentioned as C01–C09 in manuscript. **(A)** Protein. **(B)** Tryptophan. **(C)** Methionine. **(D)** β-carotene. **(E)** Phenols. **(F)** Sinapine. **(G)** Phytic acid. **(H)** Fiber. **(I)** Tannin. **(J)** GLS.

To elucidate the genetic architecture of quality traits in *B. napus* seed meal, GWAS between quality traits and genotyped SNPs was performed using the panel of 96 accessions of *B. napus* diversity fixed set. A total of 789 significant SNPs out of a total of 77,889 SNPs were found to be located on 19 chromosomes of *B. napus* with distribution over the two genomes (A and C) of *B. napus*. Majority of significant SNPs were observed for the traits sinapine (101), tryptophan (100), β-carotene (99), and protein (98) whereas a limited number of significant SNPs were reported for the traits phytic acid (49) and phenol (52). Out of 789 significant SNPs, 32 loci associated with crude protein (3), methionine (3), tryptophan (3), β-carotene (1), phenols (4), sinapine (1), phytic acid (6), crude fiber (5), tannin (3), and glucosinolates (3), respectively, were identified (Table 3). Significantly associated SNPs per trait were displayed on Manhattan plots (Figure 2). The predictive Q-Q plots show that expected distribution agrees of *p*-values have a high consistency with the observations (Supplementary Figure S2). The SNPs for crude protein content, *ASP5 (ASPARTATE AMINOTRANSFERASE 5), ASP2 (ASPARTATE AMINOTRANSFERASE 2)*, and *EMB1027 (EMBRYO DEFECTIVE 1027)*, were distributed on chromosomes A03, A10, and C02, respectively. A number of two genes, such as *MS3 (METHIONINE SYNTHASE 3)* and *CYSD1 (CYSTEINE SYNTHASE D1)* annotated on A03 and *MTO1 (METHIONINE OVERACCUMULATION 1)* envisaged on chromosome C01, were found associated with methionine content. Chromosome A10 contains two genes such as *GLU1 (GLUTAMATE SYNTHASE 1)* and *PGM (COFACTOR-DEPENDENT PHOSPHOGLYCERATE MUTASE)* and chromosome A09 showed one locus-*HEMA2* (glutamyl-tRNA reductase family protein) associated with tryptophan content. In case of β-carotene, ISPH/ *HDR (4-HYDROXY-3-METHYLBUT-2-ENYL DIPHOSPHATE REDUCTASE)* was identified on chromosome C05 at a gene distance of 32.8 kb. Significant candidate genes were predicted for antinutritional factors which include candidate *GAUT13* (*GALACTURONOSYLTRANSFERASE 13*), *UGT (UDP*-glycosyltransferase superfamily protein*), LYC (LYCOPENE CYCLASE)* and *COS1 (CORONATINE INSENSITIVE1 SUPPRESSOR 1)* on chromosomes A01, A06, A08, and C08, respectively, associated with phenols. *COS1* was envisaged at a distance of 3.59 kb from the SNP SNC_027774.2_284044517. This study also identified association of *ISPF (ISOPRENOID F)* (AT1G68970) gene on chromosome A09 with sinapine. A total of six candidate genes such as *PMT5 (POLYOL/MONOSACCHARIDE TRANSPORTER 5)* on chromosome A01, *PLDALPHA1 (PHOSPHOLIPASE D ALPHA 1)* on chromosome A05, *SAC8 (SAC DOMAIN-CONTAINING PROTEIN 8)* on chromosome A06, *PRAF1 (REGULATOR OF CHROMOSOME CONDENSATION (RCC1) FAMILY WITH FYVE ZINC FINGER DOMAIN-CONTAINING PROTEIN)* on chromosome A07, *PIP5Ks (PHOSPHATIDYLINOSITOL-4-PHOSPHATE 5-KINASE)* on chromosome A09, and *CCI1 (CLAVATA COMPLEX INTERACTOR 1)* on chromosome C02 were predicted for phytic acid. Genes involved in crude fiber synthesis pathway, i.e., *NAC073 (NAC domain-containing protein 73)* were predicted two times on chromosome C01. Functional annotation also predicted two genes *viz., BGLU45 (BETA GLUCOSIDASE 45)* and *BGLU46 (BETA GLUCOSIDASE 46)* between 2.77 and 4.75 kb on either side of the peak SNPs on chromosome C01 for lignin biosynthesis. Annotation also predicted *CYT1 (CYTOKINESIS DEFECTIVE 1)* on chromosome C03 encoding forglucose-1-phosphate adenylyltransferase family protein, an important component in cellulose biosynthesis. GWAS also allowed for recognition of three candidates such as *SOT12 (SULFOTRANSFERASE 12), SK1 (SHIKIMATE KINASE 1)*, and *UGT88A1 (UDP-GLUCOSYL TRANSFERASE 88A1)* on chromosomes A02, C07, and C08, respectively, associated with tannins. Functional annotation predicted two genes such as *DIN2 (DARK INDUCIBLE 2)* and *GSTT2 (GLUTATHIONE S-TRANSFERASE THETA 2)* on chromosome C08 and one candidate *CYP79A2 (CYTOCHROME P450 79A2)* on chromosome A10 associated with glucosinolates. Out of the two genes predicted on chromosome C08, *GSTT2* was close to SNP SNC_027774.2_32661558 at a distance of 3.9 kb. The gene expression profile of these genes was studied from Brassica EDB database (https://brassica.biodb.org/) for *B. napus*, and the results are shown in Supplementary Figure S3.

**Table 3:** Summary of the significant candidate genes of seed quality traits.

**Trait**	**Candidate gene**	**SNP IDs**	**SNP location**	**Gene distance (bp)**	**AT ID**	**Chr**.	**–log10P**	**Gene function**
Crude Protein	*ASP5*	SNC_027759.2_44220783	44220783	23664	AT4G31990	A03	3.47	Amino acid biosynthesis
	*ASP2*	SNC_027766.2_13294871	13294871	43670	AT5G19550	A10	3.92	Amino acid biosynthesis
	*EMB1027*	SNC_027768.2_8835114	8835114	33113	AT4G26300	C02	3.48	Amino acid biosynthesis
Methionine	*MS3*	SNC_027759.2_4885674	4885674	21484	AT5G20980	A03	3.04	Methionine biosynthesis
	*CYSD1*	SNC_027759.2_21536878	21536878	25775	AT3G04940	A03	3.17	Methionine biosynthesis
	*MTO1*	SNC_027767.2_23748159	23748159	8113	AT3G01120	C01	4.47	Methionine accumulation
Tryptophan	*HEMA2*	SNC_027765.2_44309263	44309263	39699	AT1G09940	A09	3.19	Tryptophan biosynthesis
	*GLU1*	SNC_027766.2_21272659	21272659	34503	AT5G04140	A10	3.07	Tryptophan biosynthesis
	*PGM*	SNC_027766.2_21272659	21272659	31690	AT5G04120	A10	3.07	Tryptophan biosynthesis
β-carotene	*HDR*	SNC_027771.2_29995244	29995244	32802	AT4G34350	C05	3.33	MEP Pathway
Phenols	*GAUT13*	SNC_027757.2_35744643	35744643	17832	AT3G01040	A01	3.24	Lignification
	*UGT*	SNC_027762.2_2096323	2096323	25421	AT3G55700	A06	4.10	Polyphenol biosynthesis
	*LYC*	SNC_027764.2_19576124	19576124	15007	AT3G10230	A08	3.59	Beta carotene biosynthesis
	*COS1*	SNC_027774.2_28404517	28404517	3591	AT2G44050	C08	3.98	Riboflavin biosynthesis
Sinapine	*ISPF*	SNC_027765.2_8996403	8996403	49451	AT1G63970	A09	3.00	MEP Pathway
Phytic acid	*PMT5*	SNC_027757.2_27740015	27740015	39548	AT3G18830	A01	3.30	Myo-inositol transport
	*PLDALPHA1*	SNC_027761.2_26966958	26966958	28955	AT3G15730	A05	3.23	Regulator
	*SAC8*	SNC_027762.2_34136832	34136832	15543	AT3G51830	A06	3.35	Phosphoinositides regulator
	*PRAF1*	SNC_027763.2_16570114	16570114	17113	AT1G76950	A07	3.12	Phosphatidylinositol binding
	*PIP5Ks*	SNC_027765.2_10864712	10864712	47184	AT1G60890	A09	3.35	Phosphatidylinositol (4,5)-biphosphate biosynthesis
	*CCI1*	SNC_027768.2_9049336	9049336	16929	AT5G65480	C02	3.22	Phosphatidylinositide binding
Crude fiber	*NAC073*	SNC_027767.2_8367432	8367432	13635	AT4G28500	C01	3.13	Cellulose and hemicellulose biosynthesis
	*NAC073*	SNC_027767.2_8367432	8367432	32549	AT4G28500	C01	3.13	Cellulose and hemicellulose biosynthesis
	*BGLU46*	SNC_027767.2_41296329	41296329	2771	AT1G61820	C01	3.24	Lignin biosynthesis
	*BGLU45*	SNC_027767.2_41336199	41336199	4750	AT1G61810	C01	3.27	Lignin biosynthesis
	*CYT1*	SNC_027769.2_22881292	22881292	22019	AT2G39770	C03	3.23	Cellulose biosynthesis
Tannin	*SOT12*	SNC_027758.2_26548840	26548840	26643	AT2G03760	A02	3.23	Flavonoids regulator
	*SK1*	SNC_027773.2_58538393	58538393	15937	AT2G21940	C07	3.08	Shikimate pathway
	*UGT88A1*	SNC_027774.2_36266109	36266109	16233	AT3G16520	C08	3.54	Flavonoid biosynthesis
GLS	*CYP79A2*	SNC_027766.2_18835337	18835337	4307	AT5G05260	A10	3.14	Glucosinolates biosynthesis
	*DIN2*	SNC_027774.2_32661558	32661558	37007	AT3G60140	C08	3.33	Glucosinolate catabolism
	*GSTT2*	SNC_027774.2_32661558	32661558	3903	AT5G41240	C08	3.33	Isothiocyanates conjugation

### Gene Pathway

**Figure 3:**
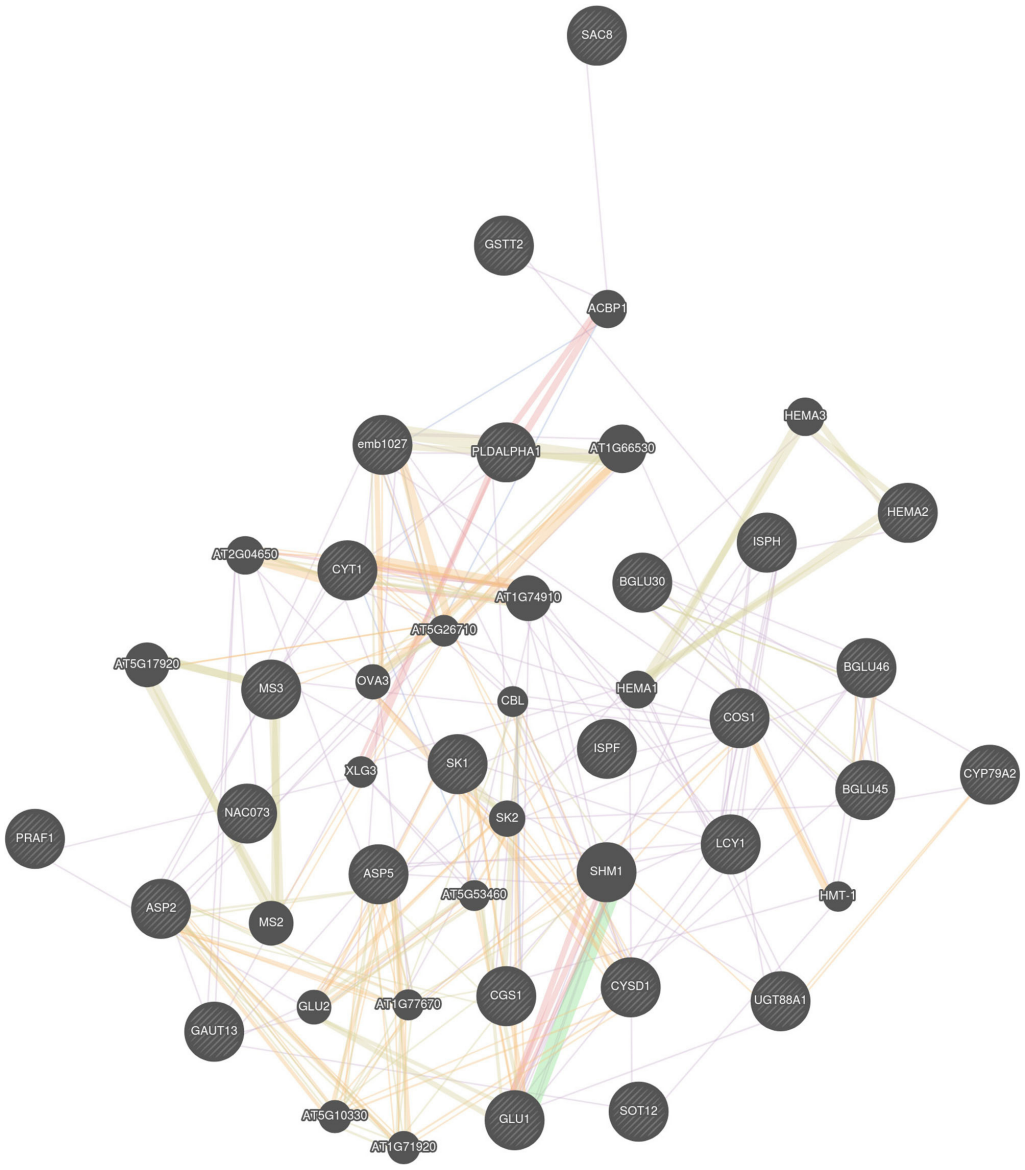
Gene pathways detected by GeneMANIA.

All the identified candidate genes were queried into GeneMANIA web server to predict their interaction, and it results into a big network showing a complex genetic relationship among them (Figure 3). Many genes are related by their functional protein domain whereas others are expressing together and physically interacting with each other to perform a similar role in cell. For example, *BGLU45* and *BGLU46*, this pair of genes have been identified on same chromosome C01 in close vicinity of a single SNP. In the gene network, they are co-expressing and physically interacting and are predicted to be responsible for affecting the crude fiber content in Brassicas. Similarly, genes identified for glucosinolates, *DIN2*, and *CYP79A2* are also co-expressing, which further gives an idea that they may be playing a role in regulation of glucosinolate pathway in plants. No direct association between the identified genes for protein was detected by GeneMANIA, but the program extended the network by associating another family member of same family, *MS2*, with *MS3 based* on the shared protein domain. A total of two of the identified genes, *ASP5* and *ASP2*, belong to the same family. According to GeneMANIA results, they are shown to share the protein domains as well. They are not found to be interacting directly with each other, but they are observed to be interacting with a same set of genes.

## Discussion

The rapeseed meal constitutes an alternative source of plant protein and holds the potential to remove malnutrition problem. However, it has limited application due to the presence of antinutritional compounds which results in extensive wastage of rapeseed meal. To make rapeseed meal fit for food and feed purpose, it is essential to improve the quality of its meal by decreasing the amount of antinutritional compounds. The development of strains with increased protein, limiting amino acids and β-carotene content coupled with low meal phenols, sinapine, phytic acid, crude fiber, tannins, and glucosinolates in seed meal is a major crop improvement goal in *B. napus*. Also, the work on genetics of these traits will promote the usage of high-quality rapeseed meal at large scale.

The outcomes of the present report showed phenotypic correlations among the ten seed meal quality traits in question. For instance, limiting amino acids (methionine and tryptophan) had significant negative correlation with protein content and significant positive correlation with each other. Based on this observation, it can be concluded that increase in protein content will be accompanied by a decrease in the levels of sulfur amino acid (methionine) and tryptophan in Brassicas. Research studies have also shown a negative relationship of seed protein concentration with methionine, tryptophan, cysteine, lysine, and threonine, whereas arginine and glutamic acid increase with seed protein concentration (Medic et al., 2014; Pfarr et al., 2018). However, it is possible to surpass the problem associated with the inverse relationship between protein concentration and sulfur containing amino acids. Studies have shown that the effect of coordinated application of nitrogen and sulfur increased the concentration of protein and essential amino acids in soybean (Moro Rosso et al., 2020), oilseed mustard (Mohiuddin et al., 2011), maize grains (Liu et al., 2021), chickpea (Chiaiese et al., 2004), and wheat and barley (de Ruiter and Karl, 2001), thereby significantly improving nutritional quality of these crops. In oilseed Brassica, the reason for this improvement could be due to the fact that nitrogen is an integral part of the protein and the protein of rapeseed contains relatively large quantities of S containing amino acids such as methionine and cystine (Gardner et al., 1985). The glucosinolate content had a significant positive correlation (*p* = 0.290) with phytic acid content, which is consistent with the previous studies done in *B. juncea* (Sharma et al., 2019). Diversity analysis results depicted the formation of ten clusters with variable number of genotypes in D^2^ analysis, which signifies the presence of genetic diversity in the germplasm. Based on the inter-cluster distances, genotypes present in clusters 4 and 9 were most diverge for the quality traits measured whereas genotypes in clusters 6 and 8 exhibited least divergence. The best genotypes could be selected based on the mean performance of the genotypes for various quality traits and inter-cluster distances. The best performing genotypes were found in clusters 4 (EC-609305, FAN-628, OCN-106, PN-45-1, PN-87-3, ZY-008) and cluster 7 (BCN-16, CHARLTON, OCN-69, PN-47-1, VCN-9). These lines had low content of antinutritional factors, high protein content, and high amount of antioxidants which could be used as parents in crossing programs for breeding rapeseed varieties.

Genome-wide association analysis, based on millions of molecular markers, is currently widely used in the analysis of complex quality traits for crops such as wheat (Yang et al., 2021), maize (Zheng et al., 2021), pea (Gali et al., 2019), soybean (Shook et al., 2021), flaxseed (Soto-Cerda et al., 2018), and Brassica (Xiao et al., 2019). Many reports addressed use of GWAS for the identification of candidate genes associated oil content (Pal et al., 2021), fatty acids (Tang et al., 2019), glucosinolate (Qu et al., 2015), and fiber fractions (Gajardo et al., 2015; Körber et al., 2016) in *B. napus*. GWAS in combination with transcriptome analysis was also explored to predict seven functional candidate genes for the seed oil content in *B. napus* (Xiao et al., 2019). However, work on the identification of significant SNPs associated with methionine, tryptophan, β-carotene, phytic acid, sinapine, etc., by GWAS has been rarely been reported, to our knowledge, and an overview of networks involved in genetic control of these traits is lacking. It is thus desirable to identify the molecular markers and candidate genes associated with these quality traits in *B. napus* which could provide valuable information about their genetic control. Hence, we investigated the genetics of seed meal quality traits through GWAS based on *B. napus* diversity panel. In this study, a total of 789 significant candidate regions were searched, of which 32 possible candidate genes for seed quality traits were predicted. We identified several genes assigned to seed meal quality traits. These were placed both on A (A01, A02, A03, A05, A06, A07, A08, A09, A10) and C (C01, C02, C03, C05, C07, C08) genome chromosomes. We obtained three candidate genes *viz., ASP5* (Wilkie et al., 1995), *ASP2* (Schultz et al., 1998), *and EMB1027* (Duchêne et al., 2005) on chromosomes A03, A10, and C02, respectively, which encode for aspartate aminotransferase 5, aspartate aminotransferase 2, and arginyl-tRNA synthetase, (class Ic) which was directly involved in the synthesis or regulation of various amino acids, building blocks of proteins. The results correspond with previous studies, reporting SNPs significantly associated with protein content on chromosome C02 of *B. napus* (Tang et al., 2019). Few reports have focused on the identification of QTL associated with total seed protein in canola (Schatzki et al., 2014; Chao et al., 2017; Behnke et al., 2018). Even fewer reports of QTL are associated with amino acid (Wen et al., 2016). Brassica quality breeding programs have made efforts to increase protein content and improve the composition of essential amino acids such as lysine (Falco et al., 1995; Wang et al., 2011) and methionine (Kohno-Murase et al., 1995; Galvez et al., 2008). On chromosome A03, we predicted two loci, *MS3* and *CYSD1*, encoding for methionine synthase 3 and cysteine synthase D1, respectively. The former was found to be involved in methionine biosynthesis (Ravanel et al., 2004), and the latter plays a role in cysteine synthesis, later required for methionine biosynthesis (Yamaguchi et al., 2000). Another significant candidate, *MTO1-*encoded pyridoxal phosphate (PLP)-dependent transferases superfamily protein, which was located on chromosome C01 has a function in methionine accumulation (Inaba et al., 1994). A total of two loci, *GLU1* and *PGM*, were annotated on the regions surrounding the significant SNP, SNC_027766.2_21272659 on chromosome A10 at a distance of 34.5 and 31.7 kb, respectively. *GLU1* encoding for glutamate synthase 1 plays a role in glutamate biosynthesis which is a part of tryptophan biosynthetic pathway (Muñoz-Nortes et al., 2017), *PGM* encoding for phosphoglycerate mutase family protein is a part of serine biosynthetic pathway, and serine is required for tryptophan biosynthesis (Chiba et al., 2012). *HEMA2* encoding for glutamyl-tRNA reductase family protein which was located on chromosome A09 also has involvement in tryptophan biosynthesis. *HDR*, annotated for the antioxidant β-carotene, encodes for 4-hydroxy-3-methylbut-2-enyl diphosphate reductase which acts as a regulator in methyl-D-erythritol 4-phosphate (MEP) pathway (Guevara-García et al., 2005). For phenol content, *GAUT13*-encoded galacturonosyl transferase 13 that is involved in lignification (Wang et al., 2013), *UGT-*encoded UDP-Glycosyltransferase superfamily protein, part of flavonoid (polyphenolic compounds) biosynthesis (Yin et al., 2017), *LYC-*encoded lycopene cyclase having a role in β-carotene biosynthesis (Cunningham et al., 1996), and *COS1-*encoded 6,7-dimethyl-8-ribityllumazine synthase involved in penultimate step of riboflavin biosynthesis (Jordan et al., 1999), were predicted using GWAS. A candidate gene *ISPF* was envisaged for sinapine on chromosome A09 and is known to be involved in non-mevalonate pathway and biosynthesized isopentenyl diphosphate and dimethylallyl diphosphate (MEP pathway) (Hsieh and Goodman, 2006). Various candidates were predicted for phytic acid and are involved in its biosynthesis pathways. *PMT5-*encoding polyol/monosaccharide transporter 5 was found involved in the transport of myo-inositol (Klepek et al., 2005). Most of the reported actions of phytic acid, including its effect on mineral absorption, have been linked to the chemical properties of the phosphate groups of myo-inositol ring (Silva and Bracarense, 2016). *PLDALPHA1* encoded for phospholipase D alpha 2 is an important regulator of phosphoinositide binding (Qin et al., 1997). Biosynthesis of phytic acid involves the myo-inositol production that involves the synthesis of compounds such as phosphoinositides, phosphatidylinositol, and so on. *SAC8* encoded SAC domain-containing protein 8, which acts as the regulators of phosphoinositides (Zhong and Ye, 2003), *PRAF1* encoding for regulator of chromosome condensation (RCC1) family with FYVE zinc finger domain-containing protein is involved in phosphatidylinositol binding (how to correlate it with phytic) (Jensen et al., 2001), *PIP5Ks* encoding for phosphatidylinositol-4-phosphate 5-kinase family protein catalyzes the synthesis of phosphatidylinositol (4, 5)-biphosphate binding (Van den Bout and Divecha, 2009) and *CCI1* encoding for CCI1; Clavata complex interactor 1 protein has a phosphatidylinositide-binding activity (Gish, 2013). A gene involved in cellulose and hemicellulose biosynthesis—*NAC073* (Hussey et al., 2011), another cellulose biosynthetic gene—*CYT1* (Lukowitz et al., 2001), lignin biosynthetic genes—*BGLU45* and *BGLU46* (Escamilla-Treviño et al., 2006; Chapelle et al., 2012) were identified on C genome for crude fiber content in our association study. Tannins are a major group of polyphenols, and various candidates were predicted for tannins in our study. *SOT12* encoding for sulfotransferase 12 is involved in regulation of flavonoids (Hashiguchi et al., 2013), *SK1*encoding for shikimate kinase 1 plays a role in shikimate pathway (Fucile et al., 2008) and *UGT88A1* encoding for UDP-glucosyl transferase 88A1 is involved in the biosynthesis of flavonoid—quercetin (Lim et al., 2004). A total of three genes were annotated for glucosinolates. *CYP79A2* encoding for phenylalanine N-monooxygenase-like protein was annotated at a gene distance of 4.3 kb and is known to be involved in biosynthesis of glucosinolates by converting L-phenylalanine to phenylacetaldoxime (Wittstock and Halkier, 2000)*, DIN2* encoding for glycosyl hydrolase superfamily protein has a function in glucosinolates catabolism and are known as putative myrosinases (Morikawa-Ichinose et al., 2020), and *GSTT2* encoding for glutathione S-transferase theta 2 is involved in the conjugation of isothiocyanates which are naturally released from glucosinolates precursors (Wagner et al., 2002).

## Conclusion

Quality improvement is the main focus of the Brassica breeding programs. Scarce information is available with Brassica breeders regarding molecular markers associated with seed meal quality traits especially for traits—β-carotene, tannin, sinapine, fiber, phytic acid, etc. The outcomes of our study helped in the identification of eleven genotypes and thirty-two candidate genes significantly associated with these seed meal quality traits of *B. napus* which would offer helpful insight to promote breeding of high-quality varieties in Brassica. Further work may involve functional validation of the identified candidate genes that will underpin the genetic systems underlying the biosynthesis of these key nutrients and antinutrients basis of seed quality in Brassica.

## Data Availability Statement

The datasets presented in this study can be found in online repositories. The names of the repository/repositories and accession number(s) can be found below: National Center for Biotechnology Information (NCBI) BioProject database under accession number PRJNA814866.

## Author Contributions

SSh designed and supervised the study. GB performed the phenotyping experiments. SSh and HK analyzed the phenotypic data and wrote the paper. SSa provided genotype by sequencing based genome assembly, SNP data, and population structure of diversity fixed set of B. napus. JA, HK, and MM conducted association mapping. SSh, HK, and JA reviewed the manuscript. All authors contributed to the article and approved the submitted version.

## Conflict of Interest

The authors declare that the research was conducted in the absence of any commercial or financial relationships that could be construed as a potential conflict of interest.

## Publisher's Note

All claims expressed in this article are solely those of the authors and do not necessarily represent those of their affiliated organizations, or those of the publisher, the editors and the reviewers. Any product that may be evaluated in this article, or claim that may be made by its manufacturer, is not guaranteed or endorsed by the publisher.

## Supplementary Material

Supplementary Figure S1 Mahalnobis euclidean^2^ distance depicting inter- and intra-cluster distance among ten clusters of 96 *B. napus* accessions by Tocher's method.

Supplementary Figure S2 Quantile-quantile (Q-Q) plots of estimated –log_10_(*p*) value from association analysis of ten seed meal quality traits using MLM model.

Supplementary Figure S3 Heatmap of candidate genes expression (RPKM value) profiling of *B. napus* germplasm on different stages.
