# Supplementary figures and images for: Genomic Regions Associated With Seed Meal Quality Traits in Brassica napus Germplasm
Source: Front Plant Sci. 2022 Jul 14;13:882766. doi: 10.3389/fpls.2022.882766 (PMC9333065; doi:10.3389/fpls.2022.882766)

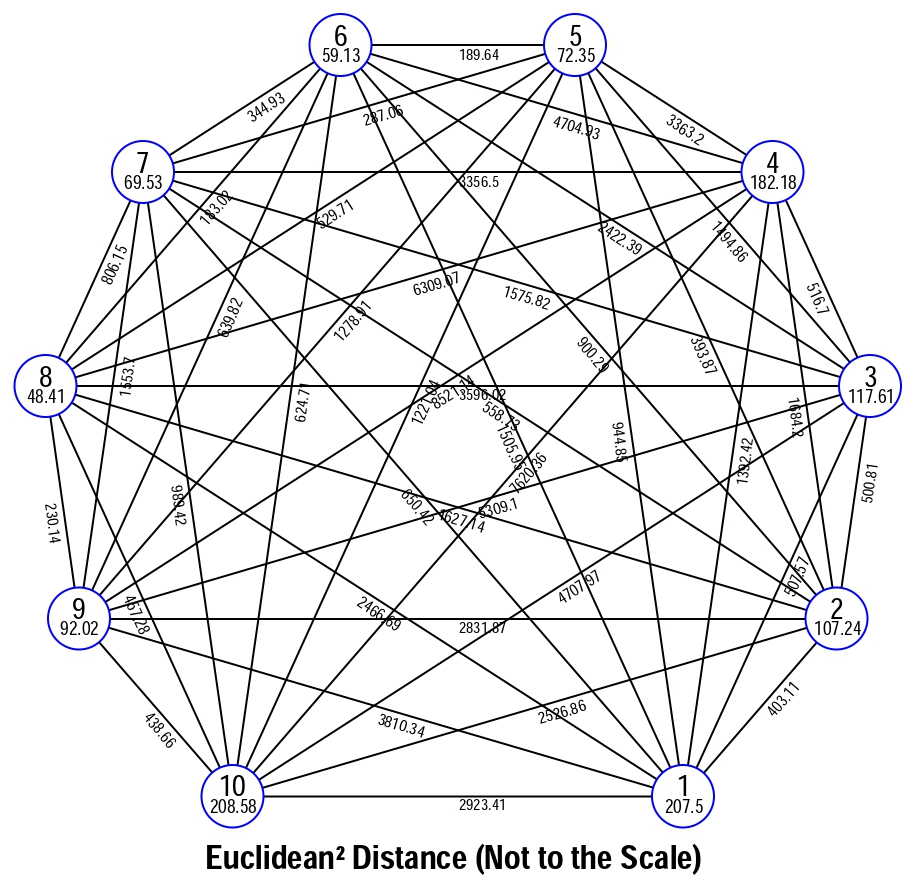

Supplement: Supplementary Figure S1 — Mahalnobis euclidean2 distance depicting inter- and intra-cluster distance among ten clusters of 96 B. napus accessions by Tocher's method. [file Image_1.JPEG]

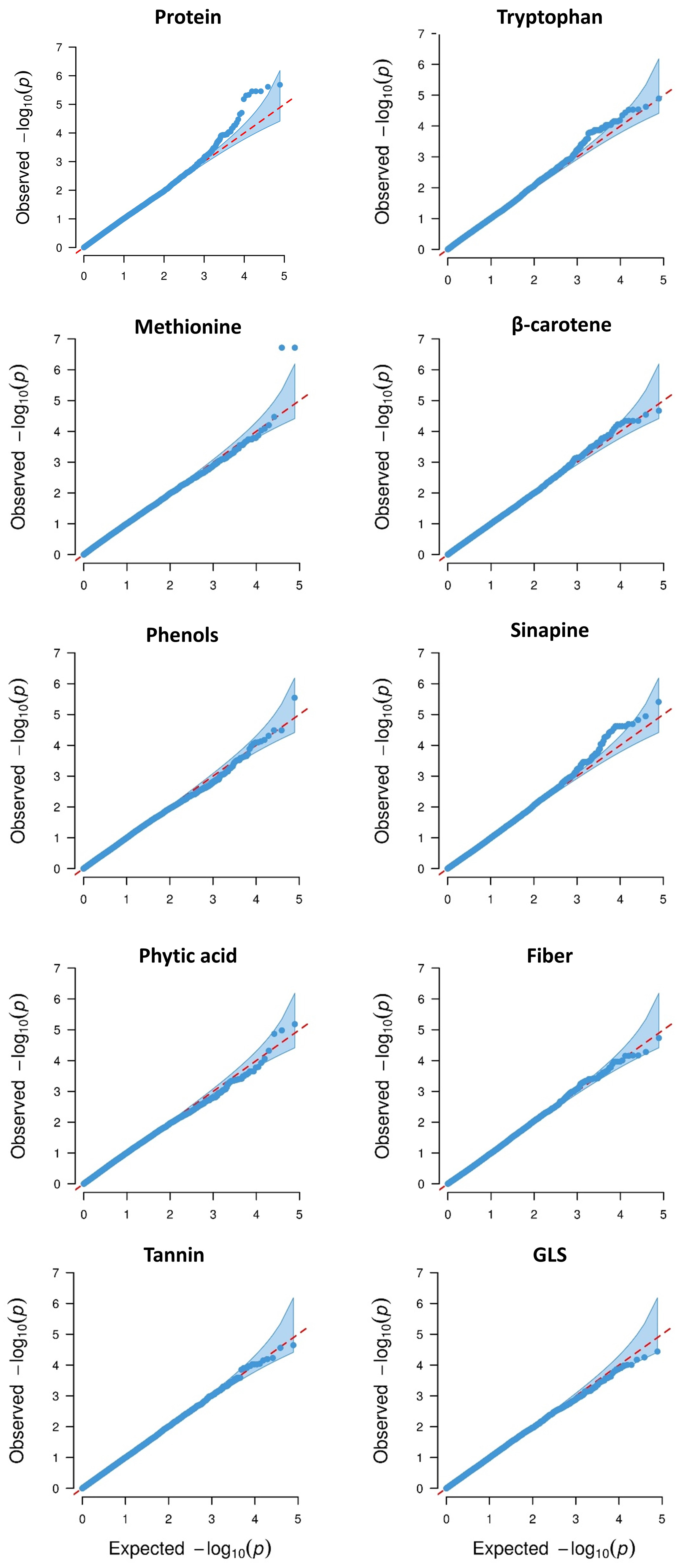

Supplement: Supplementary Figure S2 — Quantile-quantile (Q-Q) plots of estimated –log10(p) value from association analysis of ten seed meal quality traits using MLM model. [file Image_2.JPEG]

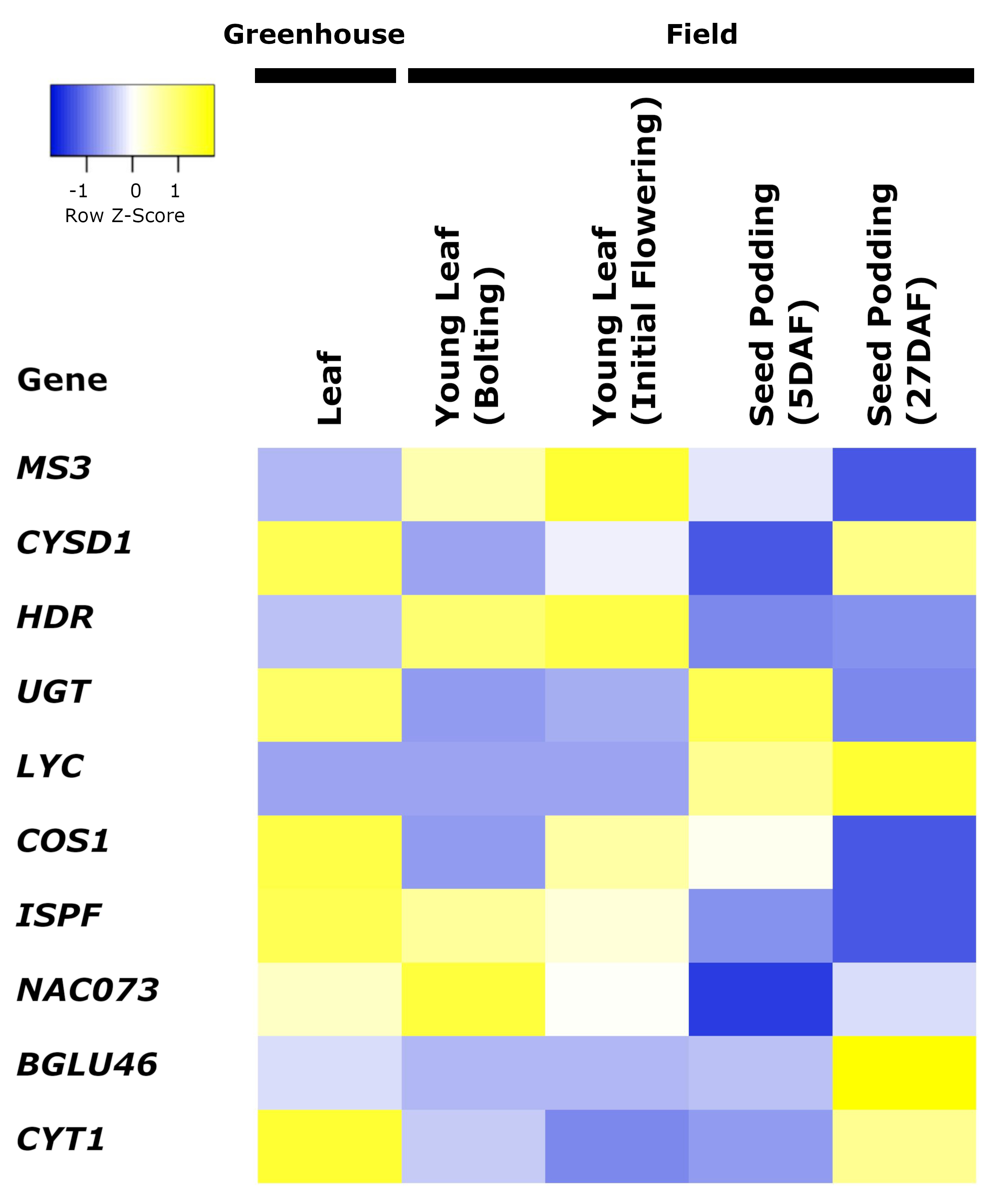

Supplement: Supplementary Figure S3 — Heatmap of candidate genes expression (RPKM value) profiling of B. napus germplasm on different stages. [file Image_3.JPEG]
